# Utilizing the apical-out enteroids *in vitro* model to investigate intestinal glucose transport, barrier function, oxidative stress, and inflammatory responses in broiler chickens

**DOI:** 10.3389/fphys.2024.1470009

**Published:** 2024-11-06

**Authors:** Peter Mann, Jundi Liu, Liang-en Yu, Ross Wolfenden, Yihang Li

**Affiliations:** ^1^ Department of Animal and Food Sciences, University of Delaware, Newark, DE, United States; ^2^ Animal Nutrition BU, Eastman Chemical Company, Kingsport, TN, United States

**Keywords:** enteroids, barrier function, inflammation, oxidative stress, broiler

## Abstract

**Introduction:**

Conventional 2D intestinal epithelial cell lines have been widely used in investigating intestinal functions, yet with limitations in recapitulating the *in vivo* gut physiology of chickens. A recently established chicken enteroid model with apical-out nature and the presence of leukocyte components represents intestinal mucosal functions. The objectives of this study were to 1) evaluate basic gut nutrient transport and barrier functions in this model and 2) identify the model’s effectiveness in studying inflammation and oxidative stress responses.

**Methods:**

Enteroids were generated from individual villus units isolated from the small intestine of Cobb500 broiler embryos. Enteroid viability, morphology, and epithelial cell markers were monitored; barrier function was evaluated based on the permeability to fluorescein isothiocyanate–dextran (FD4) with or without EDTA and lipopolysaccharide (LPS) challenges; nutrient transport was evaluated by fluorescence-labeled glucose (2NBD-G) with or without transporter blockade; the oxidative status was indicated by reactive oxygen species (ROS). Inflammatory and oxidative challenges were induced by LPS and menadione treatment, respectively. Selected marker gene expressions, including tight junction proteins (CLDN-1, CLDN-2, ZO-1, and OCCL), epithelial cell markers (Lgr-5, LYZ, and MUC-2), cytokines (IL-1β, IL-6, IL-8, IL-10, TNF-α, and INF-γ), and antioxidant enzymes (Nrf-2, catalase, and SOD), were determined by using RT-qPCR. Data were analyzed by one-way ANOVA among treatment groups.

**Results:**

Enteroid cell activity was stable from day (d) 2 to d 6 and declined at d 7. Epithelial cell marker and cytokine expressions were stable from d 4 to d 6. FD4 permeability was increased after the EDTA treatment (*P* ≤ 0.05). Transporter-mediated 2NBD-G absorption was observed, which was reduced with glucose transporter blockade (*P* ≤ 0.05). Enteroids showed classic responses to LPS challenges, including upregulated gene expressions of IL-1β and IL-6, downregulated gene expressions of ZO-1 and OCCL, and increased FD4 permeability (*P* ≤ 0.05). Enteroids showed increased ROS generation (*P* ≤ 0.05) in response to oxidative stress.

**Discussion:**

In conclusion, this apical-out enteroid model is a stable alternative *in vitro* model that exhibits intestinal barrier, nutrient transport, oxidation, and inflammation functions. With this enteroid model, we developed two challenge protocols for evaluating intestinal functions under oxidative stress and inflammation conditions.

## 1 Introduction

The intestine is a complex tissue that plays a pivotal role in maintaining health and productivity in animals by managing essential functions (Ducatelle et al., 2023), including nutrient sensing and transport to ensure efficient animal growth, as well as barrier and immune functions to protect animals from environmental antigens and pathogens (Duangnumsawang et al., 2021; Zhang et al., 2019). The optimal functioning of the intestine relies not only on the functional epithelium but also on the interaction with immune cells in the lamina propria.

Despite being an ideal model for the study of intestinal functions, the utilization of *in vivo* animal trials is constrained by factors such as high cost, length of turnaround time, and challenges in conducting mechanistic research (Gilani et al., 2021; González-González et al., 2019). *In vivo* trials often struggle to pinpoint the exact mechanisms behind observed changes, further compounded by logistical and financial constraints. Conversely, *in vitro* models offer a resolution to many of these issues, providing a cost-effective and efficient alternative for studying intestinal health, especially considering the growing interest and trend in developing alternative antibiotic products for animals (Marks et al., 2022).

Currently, various *in vitro* intestinal models are employed to emulate the *in vivo* functions of the intestine to some degree. The most prevalent among these are intestinal epithelial cell lines, which form monolayers in culture closely mimicking the function of enterocytes in the intestine. Despite their widespread use in species like humans (human colonic cell line Caco-2) and pigs (porcine jejunal cell line IPEC-J2), no equivalent *in vitro* model exists for chickens. These models are valuable for assessing the epithelial transport and supporting long-term studies due to their capacity for multiple passages. However, they do not fully replicate several key intestinal characteristics, such as the mucosal layer, immune function, and diverse cell types like goblet and Paneth cells. This emphasizes the necessity for more representative models, particularly those specifically designed for chickens (Costa et al., 2019; Sambuy et al., 2005; Sun et al., 2008; Vergauwen, 2015).

This study proposes a comprehensive *in vitro* model that represented the chicken intestine, encompassing a wide range of cell populations beyond enterocytes. This model could facilitate the investigation of essential epithelial properties, such as nutrient transport and responses to inflammatory and oxidative stress. A promising advancement in this field is the utilization of chicken intestinal organoids, also known as an enteroids model. They serve as a 3D representation of the intestine *in vitro*, with their apical-out nature depicting an orientation with the apical side of the intestine facing outward rather than its typical inward, or basal out, orientation seen *in vivo*. They exhibit a diverse cellular composition like that of the *in vivo* intestine, including the presence of leukocytes. This indicates its potential to replicate numerous functions of the chicken intestine *in vitro*, making it a promising option for further research. Although the fundamental characteristics of this model have been discussed in previous literatures (Nash et al., 2021; Nash et al., 2023), there remains a need for further exploration into its applications, particularly regarding stress responses and nutrient transport in chickens. The objective of this study is to elucidate the intestinal functions embodied by this *in vitro* model and to assess its usefulness in various research contexts.

## 2 Materials and methods

### 2.1 Enteroid isolation from chicken embryos

**FIGURE 1:**
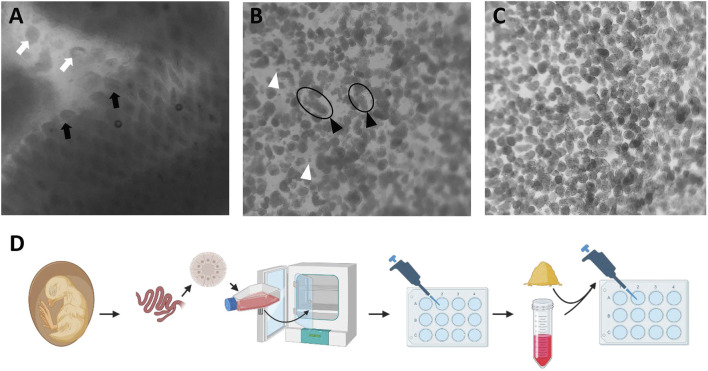
Isolation of villus units from collagenase digestion of small intestinal mucosa. **(A)** Intestinal mucosal digestion. Black arrow: villus on the mucosa; white arrow: dissociated villus unit. **(B)** Isolated villus units are passed through a 100-um cell strainer. Black arrowhead: long size villus unit; white arrowhead: individual cells. **(C)** Uniform villus units post straining through a 40–70 um cell strainer. **(D)** Schematic illustration of chicken enteroid culture and experimental design. Images obtained under an inverted microscope (Revolve, EchoTM, United States) with ×40 magnification.

All animal care procedures were approved by the University of Delaware Institutional Animal Care and Use Committee. A total of 200 Cobb500 eggs were purchased from a local hatchery and incubated at 37.5°C. At E18, chicken embryos were euthanized via decapitation. Small intestinal segments including the jejunum and ileum, from the end of the pancreas–duodenum loop to the ileal–cecal junction, were collected; cut open longitudinally; and washed with ice cold Dulbecco’s phosphate-buffered saline (DPBS, 21–031-CV, Corning™, United States). Intestinal segments were then minced to 0.5-cm pieces and digested in Dulbecco’s modified Eagle medium/nutrient mixture F-12 (DMEM/F12, 12634-010, Life Technologies™, United States) with 0.5 mg/mL collagenase (C9891, Sigma™, United States) at 37°C for 30 min. The digestion tube was gently mixed in every 10 min. After 30-min digestion, tubes were vigorously shaken to release the dissociated villus units from the gut mucosa. Samples were then filtered through serial strainers with mesh size ranging from 100 μm to 70 μm to 40 µm. The final villus units collected by using a 40-μm cell strainer were harvested by inverting the strainer and flushing with DMEM/F12 media. Villus units (40–100 μm) of uniform size were immediately cultured to enteroids or kept in freezing media, consisting of 90% DMEM/F12 and 10% dimethyl sulfoxide (DMSO and D2650, Sigma™, United States), and stored in liquid nitrogen (LN2) for later use, as shown by the procedure in Figure 1A.

### 2.2 Chicken enteroid culture

The villus units were cultured to generate enteroids following the previously published procedure (Nash et al., 2021) with modifications. Briefly, all villus units harvested from more than 50 embryos were pooled together. The villus units were then cultured in T75 culture flasks in complete media consisting of DMEM/F12 (12634-010, Life Technologies™, United States), B27 supplement (12587010, Gibco™, United States), L-glutamine (G3126, Sigma™, United States), HEPES (15630-106, Gibco™, United States), and penicillin–streptomycin (15140122, Gibco™, United States). Sphere shape apical-out enteroids were formed after 24-h culture, confirmed visually using an inverted microscope (Revolve, Echo™, United States). To avoid contamination from adherent cells and remove any non-enteroid single cells, enteroid cultures were centrifuged at 300 *g* for 5 min at 4°C degree at d 1. Enteroid pellets were resuspended in complete media and cultured in standard cell culture-treated plates at a density of 1,600 enteroids/mL. Media were changed by 50% in every 2 days until d 7 in culture. The individual wells of desired size served as an experimental unit for any functional assay. Each following functional assay consisted of four to eight replicate wells.

### 2.3 Enteroid cell activity, number quantification, and morphology

Newly formed enteroids were transferred to a 96-well plate at d 1 in culture. Each day from d 1 to d 7, a replicate batch of enteroids (n = 8 wells) was incubated with PrestoBlue (P50200, Invitrogen™, United States) for 2 h in accordance with the manufacturer’s instructions. Fluorescence was then read at 560 nm emission and 590 nm excitation in a microplate reader (SpectraMax M3, Marshall Scientific, United States). Cell activity (indicated by the mitochondrial activity measured by using the PrestoBlue kit) was calculated for the entire well of enteroid culture, including enteroids and dissociated individual cells. The data were expressed as a relative percentage value to d 1.

Cell activity,%=PrestoBlue intensity at day 1.

Enteroids were observed daily from d 1 to d 7 under an inverted microscope (Revolve, Echo™, United States) to observe their morphology and integrity with ×40 and ×100 magnification. The intact enteroids (with a clear epithelial layer surrounding) for each well of a 96-well plate (n = 8 wells) were distinguished from any broken enteroids and individual cells and were quantified under a microscope. Bright-field images were taken under the same conditions. The data were expressed as a relative percentage value to d 1.

### 2.4 Glucose transport assay

Enteroid glucose uptake were evaluated by using a fluorescent glucose 2-(N-(7-nitrobenz-2-oxa-1,3-diazol-4-yl)Amino)-2-deoxyglucose (2NBDG, N13195, ThermoFisher™, United States). At d 2, d 4, and d 6 in culture, enteroids were treated with 300 µM of 2NBDG for 30 min. To evaluate the route of glucose absorption, a sodium-glucose cotransporter 1 (SGLT1) specific glucose transporter blocker, phlorizin (PZ, 11576, Cayman Chemical™, United States), was added to enteroids at a concentration of 0.5 mM at 1 h prior to 2NBDG addition. Followed by 30-min 2NBDG incubation with or without PZ, the non-absorbed residual 2NBDG in media was removed by three washes of enteroids with ice-cold PBS at 300 x g for 5 min at 4°C. After three washes, an aliquot of the final supernatant was saved for the background reading, prior to the resuspension of the enteroid pellet. The fluorescence intensity of the final enteroid suspension was measured by a fluorescent plate reader at 465 nm emission and 540 nm excitation. The number of intact enteroids in each well was counted to normalize the 2NBDG fluorescence, as shown in the formula below. Fluorescence images were taken at the end of functional assays using an inverted microscope with ×100 magnification. The final data were expressed as a relative percentage value to d 2 non-PZ-treated control.

$$2NBDG\;fluorescence=\frac{2NBDG\;fluorescence\;intensity-Background}{Enteroid\;number}.$$

### 2.5 FD4 permeability assay

To evaluate the barrier function of the enteroid epithelial layer, the permeability of fluorescein isothiocyanate–dextran with molecular weight at 4 kDa (FD4) was measured at d 4 in culture. Barrier integrity impairment was induced by either ethylenediaminetetraacetic acid (EDTA) or lipopolysaccharides (LPS), which served as positive controls. For LPS groups, enteroids were pretreated at d 3 with 15 and 30 μg/mL LPS (Tlrl-smlps, InvivoGen™, United States) in culture media for 24 h, followed by FD4 permeability test. For EDTA groups, enteroids were pretreated at d 4 with 1 mM and 2 mM EDTA (E5391, Sigma™, United States) in ice-cold DPBS for 15 min. EDTA was then removed by washing once with ice-cold PBS. Plain ice-cold DPBS was used as the control. After EDTA pretreatment, all enteroids were plated back to the culture plate and immediately followed by the FD4 test. After 22-h incubation with LPS at 37°C with 5% CO_2_ or 15-min incubation with EDTA on ice, FD4 (46944, Sigma™, United States) was added to each well at a concentration of 2 mg/mL. This was then incubated with enteroids for 2 h post LPS treatment or 30 min post EDTA treatment. After treatment, the residual FD4 in the medium was removed by washing in ice-cold PBS, followed by 300 x *g* centrifugation at 4°C for 5 min. After washing three times, an aliquot of the final supernatant was saved for background reading, prior to the resuspension of the enteroid pellet. The fluorescence intensity of the final enteroid suspension was measured by using a fluorescent plate reader under 485 nm emission and 525 nm excitation. The number of intact enteroids in each well was counted to normalize the FD4 fluorescence, as shown in the formula below. The final data were expressed as a relative percentage value to non-treated controls. Fluorescence images were taken at the end of functional assays using an inverted microscope with ×100 magnification.

$$FD4\;fluorescence=\frac{FD4\;fluorescence\;intensity-Background}{Enteroid\;number}.$$

### 2.6 Enteroid response to inflammatory and oxidative challenges

To assess the proper response of enteroids to common experimental challenge conditions, LPS and menadione (MD, M5625, Sigma™, United States) were used for introducing inflammatory and oxidative challenges, respectively. For LPS challenge, enteroids were pretreated at d 4 with 15 and 30 μg/mL LPS (Tlrl-smlps, InvivoGen™, United States) in culture media for 6 h and then followed by RNA isolation and qRT-PCR procedures. For menadione challenge, enteroids were pretreated at d 4 with one of the following MD concentrations: 0, 12.5, 25, 50, 100, 400, or 800 µM. This was treated for 1 h, followed by CellRox (C10444, Invitrogen™, United States) reactive oxygen species (ROS) generation procedures, or pretreated with 400 µM menadione or 600 µM tert-butyl hydroperoxide (TBHP) for 6 h and then followed by RNA isolation and quantitative reverse transcription PCR (qRT-PCR) procedures.

### 2.7 Generation of ROS under oxidative challenges

To detect the response of enteroids to oxidative challenge, the generation of ROS in the enteroid culture was measured by using the commercial CellRox kit following the manufacturer’s instructions. Briefly, following 1-h incubation with menadione, CellRox (C10444, Invitrogen™, United States) was added to each well at a concentration of 5 μM. This was then incubated with enteroids for 30 min. Enteroids were then washed with ice-cold PBS three times. Enteroids were aliquoted to a black clear-bottom 96-well plate at 100 μL per well. Fluorescence was read under 485 nm emission and 528 nm excitation. Fluorescence images were taken at the end of functional assays using an inverted microscope with ×100 magnification. The final data were expressed as a relative percentage value to non-menadione-treated control.

### 2.8 RNA isolation and qRT-PCR gene expression analysis

To evaluate the basal cell activity during days in culture, enteroids were harvested at d 2, d 4, and d 6 for RNA isolation. The response of enteroids to inflammatory and oxidative challenges was analyzed in enteroids that were harvested at 6 h post challenges for RNA isolation. At isolation time, enteroids were washed by ice-cold PBS once to remove dead cell debris and individual cells. Total RNA of enteroids in each well was extracted by TRIzol™ reagent (15596026, Thermo Fisher Scientific™, United States), according to the manufacturer’s instructions. After isolation, total RNA concentration was measured through NanoDrop (13400525, Thermo Fisher Scientific™, United States). The cDNA was synthesized by using a commercial kit (K1672, Thermo Fisher Scientific™, United States). qRT-PCR was performed in a QuantStudio™ 3 Real-Time PCR Detection System (Applied Biosystems) with 2x SYBR qPCR Mix (A25742, Applied Biosystems™, United States). The comparative Ct method (2^−ΔΔCT^ method) was used to quantify gene expressions relative to the untreated control or d 2 enteroid control, with the gene ribosomal protein P0 (RPL0) and ribosomal protein P13 (RPL13) serving as internal controls. All primers were designed using the NCBI Primer-BLAST tool, ensuring specificity and optimal binding to the target sequences. Primers are listed in Table 1.

**TABLE 1:** List of primers.

Gene	Full name	Accession no.	Forward	Reverse	Amplicon
RPL-0	Ribosomal protein subunit P0	NM_204987	TTGGGCATCACCACAAAGATT	CCCACTTTGTCTCCGGTCTTAA	83
RPL-13	Ribosomal protein L13	NM_204999	TCGTGCTGGCAGAGGATTC	TCGTCCGAGCAAACCTTTTG	71
Lgr-5	Leucine rich repeat-containing G protein-coupled receptor 5	XM_425441.5	ACGTCTTGCAGGAAATGGCT	TTGGCATCCAGGCGTAGAG	159
LYZ	Lysozyme	NM_205281	GACGATGTGAGCTGGCAG	GGATGTTGCACAGGTTCC	225
MUC-2	Mucin 2	XM_040673077.2	ATTGAAGCCAGCAATGGTGT	TTGTTGGCCTTGTCATCAAA	125
CLDN-1	Claudin 1	NM_001013611.2	GCGGGGGACAACATCGTGAC	AGACCCAGGAGTATGGCGGC	170
CLDN-2	Claudin 2	NM_001277622.1	GACGTGAACCATTCGCAGTCC	GTTTTGTGAGGGCACAGGCA	151
ZO-1	Zonula occludens-1	XM_015278981.2	TGAAGGACCCCAGTGACACG	CACTCATGGCTGGGAGCGTA	75
OCCL	Occludin	NM_205128.1	TCATCGCCTCCATCGTCTAC	TCTTACTGCGCGTCTTCTGG	240
IL-1β	Interleukin 1 beta	NM_204524.1	TGCCTGCAGAAGAAGCCTCG	CTCCGCAGCAGTTTGGTCAT	137
IL-6	Interleukin 6	NM_204628.2	GCAGGACGAGATGTGCAAGA	ACCTTGGGCAGGTTGAGGTT	84
IL-8	Interleukin 8	NM_205498.1	AGCTGCTCTGTCGCAAGGTA	GCTTGGCGTCAGCTTCACATC	124
IL-10	Interleukin 10	NM_001004414.2	GTCACCGCTTCTTCACCTGC	TCCCGTTCTCATCCATCTTCTCG	84
TNF-α	Lipopolysaccharide-induced TNF factor	NM_204267.1	CCCTACCCTGTCCCACAACC	TGGGCGGTCATAGAACAGCA	150
INF-γ	Interferon gamma	NM_205149.1	ATGTAGCTGACGGTGGACCT	GCGGCTTTGACTTGTCAGTGT	135
TLR-4	Toll-like receptor 4	NM_001030693.1	CCTGGGTCTAGCAGCCTTCC	TGGCCCAGATTCAGCTCCTG	129
Nrf-2	Nuclear factor erythroid 2-related factor 2	NM_001396903.1	TCCTGGTGCAGTCTTCTGTGA	TCTCCCGCTCTTTCTGGAGC	193
Catalase	Catalase	NM_001031215.2	GACGGGCCAATGTGTGTGTC	TGAGATAGAAGTCTCGCACCTGAG	181
SOD-1	Superoxide dismutase 1	NM_205064.1	CCGGCTTGTCTGATGGAGAT	TGCATCTTTTGGTCCACCGT	125
SOD-2	Superoxide dismutase 2	NM_204211.2	GGCGCTGGCAAAAGGTGATGTT	TTGCGAAGGAACCAAAGTCACG	176
SGLT-1	Sodium glucose cotransporter	XM_415247.4	GCCATGGCCAGGGCTTA	CAATAACCTGATCTGTGCACCAGTA	71
GLUT-2	Glucose transporter 2	NM_207178.1	ATGACGGTTGGACTTGTGCT	CAATGAAGTTGCAGGCCCAG	202
ALPi	Alkaline phosphatase intestinal	XM_003641761.5	AGTGTGCACCCATAGACAGC	AGTCCATGCCCAGGATTTGG	76
CHGA	Chromogranin A	XM_015287755.2	AAGCCAACACTGATGAAGAAGG	CTGAGGTGAGTACTGGGAGC	70
PYY	Peptide YY	NM_001361182.1	ACATCAACCTGGTCACGCGG	CGATGGGCTGCACTGACACT	75

### 2.9 Statistical analysis

All data were shown as means ± standard error (SE). Individual wells containing 80 to 200 enteroids each were used as experimental units. For each enteroid viability and functional assay, no fewer than six wells were used as replicates. Significance between treatments or chemical dosages was determined by one-way ANOVA and multiple comparisons with Tukey’s test adjustment in GraphPad 10.0. For the glucose transport assay, a two-way ANOVA was performed to analyze the effects of culture days, glucose transport blockade, and their interaction. The *p* ≤ 0.05 was considered significant.

## 3 Results

### 3.1 Chicken enteroid isolation, culture, and morphology

**FIGURE 2:**
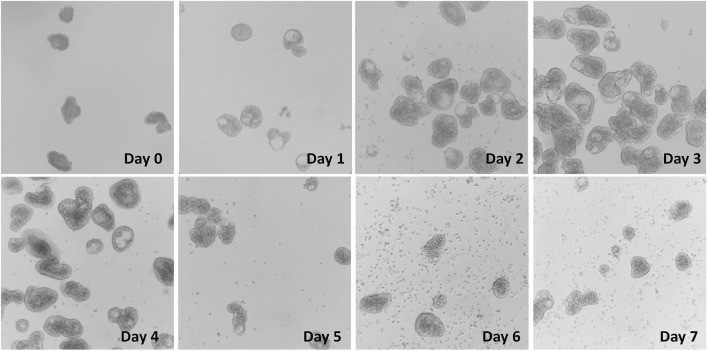
Enteroid formation and culture from villus units in FOM media at d0 to d7. Images obtained under an inverted microscope (Revolve, EchoTM, United States) with ×40 magnification.

After collagenase digestion, the intact villus units including the epithelial cell layer and lamina propria cells found beneath were dissociated from the intestinal mucosa (Figure 1A). Tissue debris was removed after straining with a 100-μm cell strainer, which produced a mixture of small and large villus units and individual dissociated cells (Figure 1B). The final isolation of villus units was conducted by using a 70-µm strainer which produced villus units of uniform size, and also by using a 40-µm strainer and invert collecting, which removed the individual cells (Figure 1C). Once incubated in floating organoid media (FOM), the apical-out enteroids were formed after 24 h culture. Significant changes in morphology were observed from d 0 to d 1, with the smooth cell membrane and spherical shape of the enteroids becoming more apparent (Figure 2). Starting at d 2, the enteroids show a healthy morphology, depicted by a clear spherical shape with a smooth outer border. The morphology of enteroids was not changed significantly from d 2 to d 7, and they were generally consistent in size over time. The cellularity in the epithelial layer of enteroids was gradually decreased, while the individual cells that were dissociated from enteroids gradually increased during the days of culture and were observed from d 6 in culture (Figure 2).

### 3.2 Chicken enteroid viability

**FIGURE 3:**
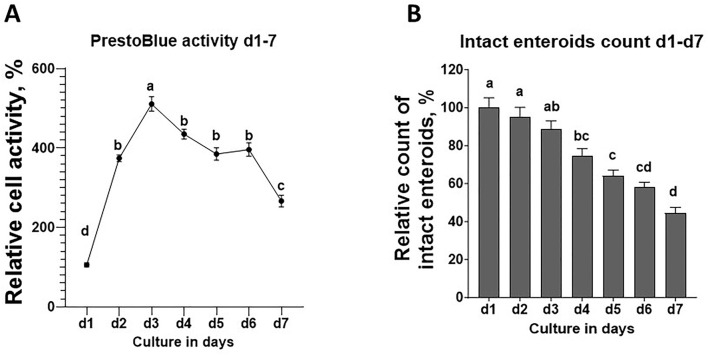
Enteroid cell activity and intact enteroid analysis from d1 to d7 in culture. **(A)** Cell activity measured by PrestoBlue analysis. **(B)** Number of intact enteroids. Measurements of all other days were normalized to d1 (100%) as the relative viability or relative count. Data are expressed as the mean ± SEM from at least six replicate wells. Different letters indicate *p* ≤ 0.05 among culture days.

To establish challenge protocols for this model, it is essential to assess the viability and identify the characteristics of chicken enteroids at different stages of culture. The enteroid culture contains the intact enteroids and the individual cells that were dissociated from unhealthy enteroids. The cellularity and mitochondria metabolism of the enteroid culture, measured by the PrestoBlue kit, were increased significantly during the first 48 h in culture, peaked at d 3, and gradually decreased till d 7 (Figure 3A). Within each enteroid culture, approximately 150–200 intact enteroids per well were cultured at d 1. The overall number of intact enteroids was gradually declined after 24 h of culture and was significantly decreased by ∼25% at d 4 and ∼55% at d 7 compared with d 1 (Figure 3B). These data indicated that the enteroid structure had gradually broken down during the culture processing, while individual cell activities remained relatively stable.

### 3.3 Chicken enteroid gene marker characterization

**FIGURE 4:**
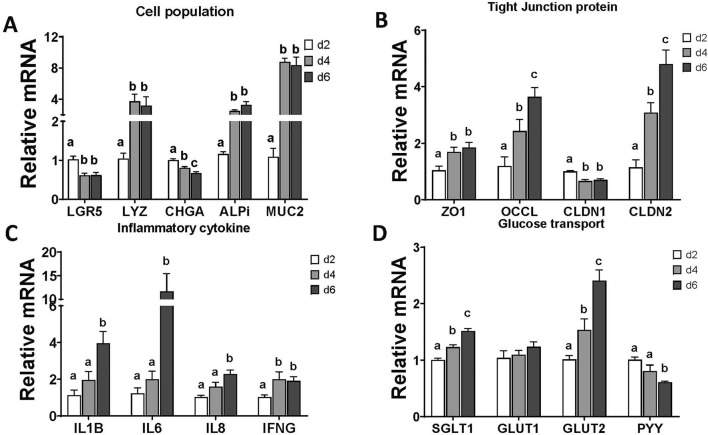
Evaluation of enteroid gene expression at d2, d4, and d6 in culture. **(A)** Gene expression of selected markers of epithelial cell populations. **(B)** Gene expression of selected tight junction proteins. **(C)** Gene expression of selected proinflammatory cytokines. **(D)** Gene expression of selected glucose transporters and endocrine hormone. Data are presented as the mean ± SEM from at least six replicate wells. Different letters indicate p ≦ 0.05 among culture days.

The mRNA expression of gene markers of the epithelial cell population was significantly changed during the days in culture. The expression of the stem cell marker, leucine-rich repeat-containing G-protein-coupled receptor 5 (Lgr-5), was significantly decreased at d 4 and d 6 compared with the expression in d 2. Similarly, the expressions of other differentiated epithelial cell markers, including Paneth cells (lysozyme, LYZ), enterocytes (alkaline phosphatase intestinal, ALPi), and goblet cells (mucin 2, MUC-2), were significantly increased at d 4 and d 6. The significant decrease in expression of enteroendocrine cell marker (chromogranin A, CHGA) was continued in d 4 and d 6 (Figure 4A).

For the structural markers in enteroids, gene expressions of the tight junction proteins, such as zona occludens 1 (ZO-1), occludin (OCCL), and claudin 2 (CLDN-2), were significantly increased starting at d 4 in enteroid culture (Figure 4B). In contrast, the expression of claudin 1 (CLDN1) was decreased at d 4 and d 6 compared with the expression in d 2.

The basal expression of enteroid functional markers, such as inflammatory cytokines and glucose transporters, was changed during days of culture. All of the selected cytokine gene expressions were upregulated at d 4 or d 6 in culture compared with d 2 (Figure 4C). The expressions of epithelial specific glucose transporters, SGLT1 and glucose transporter 2 (GLUT2), were upregulated significantly from d 4 to d 6, while the expressions of the general cell glucose transporter glucose transporter 1 (GLUT1) were not changed. The expression of enteroendocrine cell hormone peptide YY (PYY) was downregulated at d 6 significantly (Figure 4D). These data suggested increased cell maturation over the days in culture.

### 3.4 Glucose uptake in chicken enteroids

**FIGURE 5:**
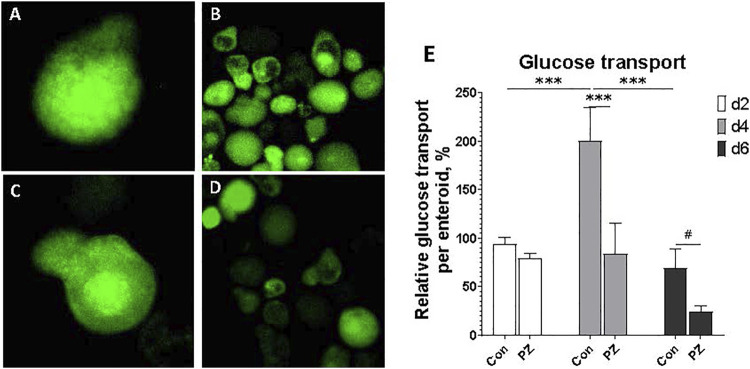
Evaluation of glucose transport in enteroids at d2, d4, and d6 in culture. **(A, B)** 2NBDG retention in the enteroids pretreated with DMSO control at ×100 and ×400 magnification, respectively. **(C, D)** 2NBDG retention in the enteroids pretreated with SGLT1 glucose transporter blocker phlorizin 0.5 mM at ×100 and ×400 magnification, respectively.**(E)** Data are presented as the mean ± SEM from at least six replicate wells. Two-way ANOVA showed culture days effect *P* ≦ 0.05, blocked effect *P* ≦ 0.05, interaction *p* = 0.06. # indicates *P* ≦ 0.10. * indicates *P* ≦ 0.05, and *** indicates *P* ≦ 0.0001 compared between PZ and Con groups, or among culture days in Con groups.

A major function of the small intestine is the uptake of nutrients across the apical membrane. To evaluate glucose transport function of chicken apical-out enteroids, a fluorescent glucose probe, 2NBDG, was used. After 30-min incubation with 2NBDG, the fluorescence signal was detected inside of each enteroid (Figure 5A–D). To identify whether this 2NBDG uptake was routed through the epithelial SGLT1 glucose transporter, the enteroids were pretreated with a specific SGLT1 blocker phlorizin (PZ). Most enteroids exhibited a reduction in the overall fluorescence intensity, with a particularly dimmed surface and an unchanged core area (Figures 5C, D) compared to the control (Figures 5A, B). Significant treatment effects and culture day effects were detected, without interaction (*p* = 0.06). The overall 2NBDG absorption per enteroids was peaked at d 4 in culture and was significantly reduced in the PZ group (Figure 5E). These data indicated that the chicken enteroids absorb glucose from media via SGLT1 transporter, and such absorption peaked at d 4 in culture.

### 3.5 Epithelial barrier function in enteroids

**FIGURE 6:**
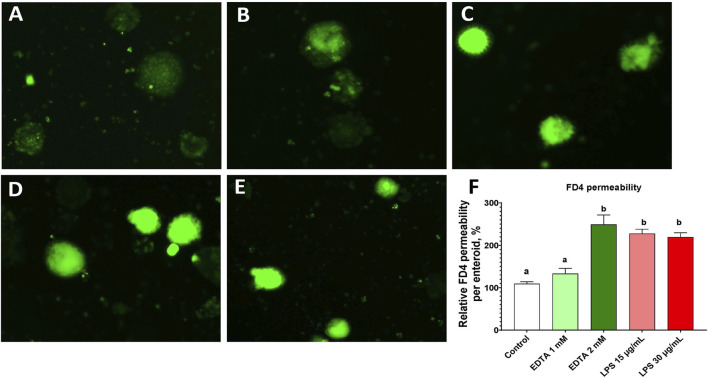
Evaluation of FD4 permeability in enteroids at d4 in culture. (A ∼ E) FD4 fluorescence retention in the enteroids pretreated with PBS control **(A)**, EDTA 1 mM **(B)**, EDTA 2 mM **(C)**, LPS 15 μg/mL **(D)**, and LPS 30 μg/mL **(E)**. **(F)** Normalized FD4 permeability relative to the PBS control. Data are presented as the mean ± SEM from at least six replicate wells. Different letters indicate *P* ≦ 0.05 among treatments.

Gut barrier function is important for separation of the luminal and basal components of the intestine. The FD4 molecule leaked through epithelial tight junctions and remained in the enteroids (Figure 6). With barrier integrity disruptions that were induced by pretreatment of EDTA and LPS, the permeability of FD4 significantly increased compared with those of the control (Figure 6F). Comparing LPS treatments of 10 μg/mL to 30 μg/mL, no major difference was seen. These results suggested that these organoids have a functional epithelial barrier and that both EDTA and LPS can significantly alter this barrier’s function.

### 3.6 Chicken enteroid mRNA expression under inflammatory challenge

**FIGURE 7:**
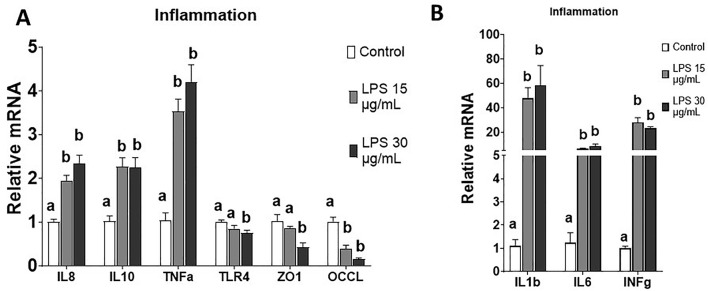
Evaluation of enteroid gene expression in response to inflammatory challenge at d4 in culture. **(A, B)** Gene expression of selected markers of proinflammatory cytokines and tight junction proteins with or without LPS challenges. Data are presented as the mean ± SEM from at least six replicate wells. Different letters indicate *P* ≦ 0.05 among treatments.

Addition of LPS to chicken enteroids resulted in significantly reduced tight junction expressions of ZO-1 and OCCL (Figure 7A). On the other hand, both 15 and 30 μg/mL of LPS induced significantly increased expression of all selected inflammatory markers, including interleukin 1 beta (IL-1B); interleukins 6, 8, and 10 (IL-6, IL-8, and IL-10, respectively); tumor necrosis factor (TNF-α); and interferon gamma (INF-g). The gene expression of toll-like receptor 4 (TLR4) was significantly decreased under 30 μg/mL of LPS. These results indicate that LPS treatment reduced enteroid barrier function and increased inflammatory response, which was similar with the LPS response in intestinal tissue.

### 3.7 Chicken enteroid response to menadione-induced oxidative challenge

**FIGURE 8:**
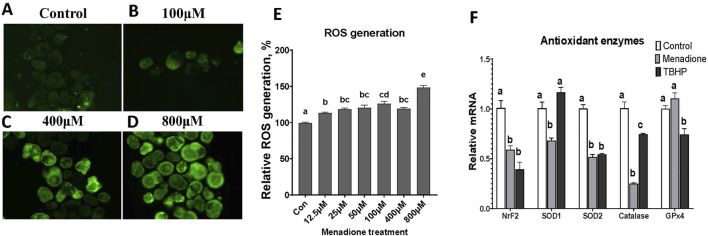
Evaluation of enteroids in response to oxidative challenge at d4 in culture. **(A–D)** ROS generation in enteroids pretreated with DMSO control, 100 μM, 400 μM, and 800 µM menadione, respectively. **(E)** Relative ROS quantification to the control. **(F)** Gene expression in enteroids pretreated with DMSO control, 400 µM menadione, or TBHP. Data are presented as the mean ± SEM from at least six replicate wells. Different letters indicate *P* ≦ 0.05 among treatments.

Menadione at increasing doses from 0μM (Con) to 800μM were used to stimulate oxidative challenge in enteroids at d 4 in culture. Significant increase in ROS generation was detected at any concentration of MD compared to control (Figure 8E). The greatest increase was seen with the 800 µM treatment, though at this concentration, the menadione started to induce turbidity within the enteroid culture. The doses of 400 µM of MD and 600 µM TBHP were selected for stimulating oxidative stress for RT-PCR analysis. Both MD and TBHP reduced gene expressions of nuclear factor erythroid 2-related factor 2 (NrF-2), superoxide dismutase 2 (SOD-2), and catalase post 6 h of treatment (Figure 8F), while only MD induced reduction in superoxide dismutase 1 (SOD-1) mRNA expression. These results suggested that enteroids generated ROS and utilized the antioxidant enzyme defense under oxidative challenge.

## 4 Discussion

In this study, we recapitulated the apical-out chicken enteroid model originally developed by Nash et al. (2023). To explore advanced applications of this model for studying intestinal functions, we evaluated optimal protocols for various functional assays, including glucose transport, barrier permeability, and responses to inflammatory and oxidative stressors. Given the stable embryonic environment, free from the influences of feed and microbiome, there are likely reduced variations across small intestine segments. Therefore, the pooled embryonic intestinal tissue used in this study is considered a reliable and homogeneous sample of the chicken small intestine. Moreover, in the embryonic intestine mucosa, the crypt fission capacity and invagination are not fully developed (Uni et al., 2000); thus, the major portion of our isolation focused on the villus units. General collagenase digestion randomly dissociates the intestinal mucosa, generating villus units of various lengths. By using cell straining, we standardized the size of these villus units and produced relatively homogeneous enteroid morphologies in culture (Figures 1, 2). As observed previously (Nash et al., 2021), these apical-out enteroids contain lamina propria cells encapsulated within a sealed epithelial layer, preventing the enteroids from being propagated in culture. We further assessed the enteroid cell activity over days in culture. As shown in Figure 2, over the course of the culture, some cells dissociated from the enteroids, resulting in a mixture of intact enteroids and individual cells. The overall cell mitochondrial activity increased, peaking at d 3 in culture (Figure 3A), indicating an increase in stable cellularity up to d 6. However, the number of intact enteroids continued to decline from d 3 (Figure 3B). It is interesting to observe the discrepancy between individual cell activity and intact enteroids over the course of days in the same culture. It appears that enteroids can dissociate into individual cells without losing cell activity. To fully evaluate enteroid function, we focused on the number of intact enteroids and excluded individual cells from all functional assays and gene expression analyses in this study. To determine the optimal culture duration for this enteroid model, we assessed the expression of key genes of interest and their dynamic changes from day 2 to day 6 in culture. Most marker genes for epithelial cell types were upregulated as the culture progressed. Similarly, tight junction proteins and inflammatory cytokines showed increased expression (Figure 4). These findings indicate that a mature and stable epithelium is achieved between days 4 and 6 in culture. Therefore, to effectively use this model for functional assays, such as assessing nutrient absorption (particularly glucose), barrier integrity, and responses to inflammatory and oxidative stress, the experimental challenges and measurements should be applied within this time frame.

Glucose absorption in the intestinal epithelium primarily relies on the SGLT1 transporter, along with paracellular diffusion (Hantzidiamantis et al., 2024; Lizák et al., 2019). In this study, we used a fluorescent d-glucose analog, 2-[N-(7-nitrobenz-2-oxa-1,3-diazol-4-yl) amino]-2-deoxy-d-glucose (2NBDG), as a glucose analog to assess overall glucose absorption in the enteroids (Hamilton et al., 2021; Zou et al., 2005; Chen et al., 2015; Leira et al., 2002; O’Neil et al., 2005). Before conducting the 2NBDG assay, the enteroids were pretreated with phlorizin, an SGLT1 blocker (Ehrenkranz et al., 2005; Ogata et al., 2014), to confirm the involvement of SGLT1-mediated glucose transport. As the epithelial cells matured over the course of the culture, glucose transport increased and peaked on day 4. The blockade of the SGLT1 transporter by phlorizin significantly reduced glucose absorption on days 4 and 6, confirming that partly mediated the glucose transport was mediated by SGLT1 (Figure 5). The increased expression of tight junction proteins and the SGLT1 transporter from day 2 to day 6, as shown in Figure 4, suggests that SGLT1-mediated glucose transport became functional and fully mature by day 4. In contrast, glucose paracellular diffusion, likely due to an immature barrier, seemed to be predominant on day 2 and declined as the barrier matured on day 6. It is interesting that glucose transport begins to decline on day 6. Given the rapid turnover of epithelial cells in the small intestine, the lifespan of a mature cell in chickens may be less than 6 days. Further investigation is needed to fully understand the epithelial cell population and maturation in enteroid cultures. Overall, our data suggest that chicken enteroids can effectively model nutrient transport *in vitro*, with the optimal day for glucose transport being day 4.

In this study, the response of enteroids to an inflammatory challenge was evaluated, focusing on LPS-induced changes in barrier integrity and gene expression. Barrier integrity was assessed using FD4 permeability (Baxter et al., 2017; Schoultz et al., 2020). Two well-established barrier disruptors were used to reduce barrier integrity. LPS activates epithelial and immune cells, leading to intestinal barrier disruption (Ghosh et al., 2020; Kim et al., 2021), while EDTA disrupts cell–cell adhesion (Pell et al., 2021; Bardenbacher et al., 2019; Liu et al., 2021; den Daas et al., 2022). Under normal control conditions, a small amount of FD4 molecules leaked paracellularly from the media into the center of the enteroids (Figure 6A). This permeability increased dramatically when the barrier integrity was disrupted by 15 and 30 μg/mL LPS and 2 mM EDTA (Figure 6B–E), without a reduction in the number of enteroids (data not shown). This LPS-induced barrier disruption was in line with the gene expression data, which showed upregulation of proinflammatory genes and downregulation of key tight junction genes. (Figure 7). The need for a high dose of LPS stimulation may reflect the LPS resilience nature of the chicken species (Berczi et al., 1966; Warren et al., 2010). Our data successfully established a protocol for evaluating barrier function and modeling inflammatory challenges in chicken enteroids.

Another challenge of interest was induced by an oxidant to evaluate the enteroids’ response to oxidative stress. Hydrogen peroxide (H₂O₂) is commonly used as an inducer because of its natural role in the body as a reactive oxygen species (ROS) (Andrés et al., 2022; Lennicke et al., 2015). However, it is challenging to use as high concentrations are required to achieve the desired oxidative effects (Ransy et al., 2020). Menadione can penetrate the cell membrane and interfere with the mitochondrial redox cycle, leading to increased ROS production. Therefore, we used menadione for its ability to induce strong oxidative effects at lower concentrations compared to H₂O₂ (Goffart et al., 2021). Of the tested concentrations, MD at 800 µM generated the greatest amount of ROS in the enteroids (Figure 8). However, we found that at this concentration, a great deal of turbidity was present in the enteroids, which could potentially affect downstream analysis. As such, we determined 400 µm as an ideal concentration of MD use in the enteroids. Downregulation of antioxidant enzyme gene expression was also observed under oxidative stress (Figure 8). Overall, this enteroid model demonstrated a functional response to common intestinal inflammatory and oxidative stress conditions.

*In vitro* models like Caco-2 and IPEC-J2 cell lines are commonly used to study intestinal physiology but have limitations in fully replicating the properties of the *in vivo* intestine, particularly due to their cancer origins and the lack of well-studied models in other species, including poultry (Iglesias et al., 2020; Chen et al., 2023; Langerholc et al., 2011). More advanced models, like intestinal organoids, offer greater complexity, incorporating multiple cell types and better representing the *in vivo* intestine. Organoids are useful for studying rare cell types, microbial interactions, and diet effects but are typically basal-out, limiting access to the luminal side. The development of apical-out organoids, with the luminal surface facing outward, has addressed this limitation, enabling better study of nutrient absorption, barrier integrity, and host–pathogen interactions across various species (Taelman et al., 2022; Boonekamp et al., 2020; Puschhof et al., 2021). Additionally, these organoids include a leukocyte component, enhancing their relevance for studying gut function (Nash et al., 2023).

The apical-out enteroid model enables *in vitro* study of the chicken intestine, providing a platform to investigate mechanisms using the previously mentioned protocols. Functional assays measuring responses to LPS and menadione (MD) are crucial for understanding how the intestine reacts to inflammatory and oxidative stress, respectively. Additionally, the model’s nutrient transport functions allow for the study of various essential nutrients beyond glucose. This makes it a superior model for examining luminal factors such as feed additives and microbiota, helping assess their effects on gut inflammation and oxidation before *in vivo* trials. Moreover, co-cultures with pathogens or microbes could offer valuable insights into the interactions between the gut and host microbiome.

## 5 Conclusion

In this study, we present evidence supporting the effectiveness of apical-out chicken enteroids as an *in vitro* model for studying the chicken intestine. Our developed protocols demonstrate their capability and efficacy in assessing intestinal function in the presence of inflammatory and oxidative stress, as well as evaluating nutrient transport.

## Data Availability

The raw data supporting the conclusions of this article will be made available by the authors, without undue reservation.
